# Metastatic adult Wilms' tumor managed by chemotherapy, immunotherapy and target therapy: a case report

**DOI:** 10.2144/fsoa-2023-0156

**Published:** 2024-05-16

**Authors:** Qian Zhao, Qi Xiong, Qi Song

**Affiliations:** 1 Department of Oncology, Dandong First Hospital, 118000, Liaoning, China; 2 Department of Oncology, Chinese PLA General Hospital, 100080, Beijing, China

**Keywords:** Adult Wilms' tumor, chemotherapy, immunotherapy, VEGF-A-targeted therapy

## Abstract

Wilms' tumor is a rare type of tumor in adult. Herein, we reported a case of 37-year-old female with adult Wilms' tumor (AWT) admitted in our institution. After a multidisciplinary team discussion, she underwent receiving immunotherapy plus chemotherapy and VEGF-targeted therapy. The tumor got smaller obviously after eight cycles of treatment. Our present case suggested that immunotherapy and anti-angiogenesis combined with chemotherapy is promising new approach for treating AWT. Moreover, we review the literatures reporting AWT with the purpose to improve the understanding of AWT treatment.

## Plain language summary

A 37-year-old woman discovered a huge renal mass with multiple lymph node metastases. After ultrasound-guided needle biopsy of tumor tissue in the right kidney, she was found to be a rare adult Wilms' tumor. After a multidisciplinary team discussion, she underwent systemic therapy. Then, we gave her two cycles of treatment, as the tumor got smaller. Then, we continued to give her six cycles of treatment. Now, she is in good condition.

## Abstract (plain language summary)

Adult Wilms' tumor (AWT) is a rare neoplasm, majority of AWT was diagnosed with advanced stage.

AWT remains poor and there is no standard care of treatment for AWT and treatment has not been thoroughly explored.

A stage IV AWT patient receiving immunotherapy plus chemotherapy and VEGF-targeted therapy.

The patients achieved partial response after two cycles of treatment. At the final follow-up in April 2023, the patient achieved a PFS of 6 months.

Nephroblastoma in adult, also known as adult Wilms' tumor (AWT), is a rare neoplasm, incidence of which is 0.2 case per million per year [1–6]. Patients with AWT are often diagnosed with advanced stage and associated with poorer survival. AWT responds poorly to conventional chemotherapy and is commonly recurred. Moreover, due to the extremely low incidence, no systemic clinical research has been generated for AWT. Therefore, there is still no established standard treatment for AWT. To share the experience of treatment in our institution, we presented a case of a 37-year-old female with AWT.

## Case presentation

A 37-year-old female patient presented with abdominal distension and abdominal mass for 2 months, no abdominal pain and hematuresis, was admitted to department of oncology in Chinese PLA General Hospital on 7 September 2022. Ultrasonography of abdomen on admission revealed a hypoechoic mass in the right kidney. Abdominal magnetic resonance imaging (MRI) revealed a space-occupying lesion in the right kidney with multiple lymph node metastases compressing the right renal vein and inferior vena cava (Figure 2A). A computed tomography scan (CT-SCAN) revealed multiple mediastinum lymph node metastasis (Figure 2B).

**Figure 1.**
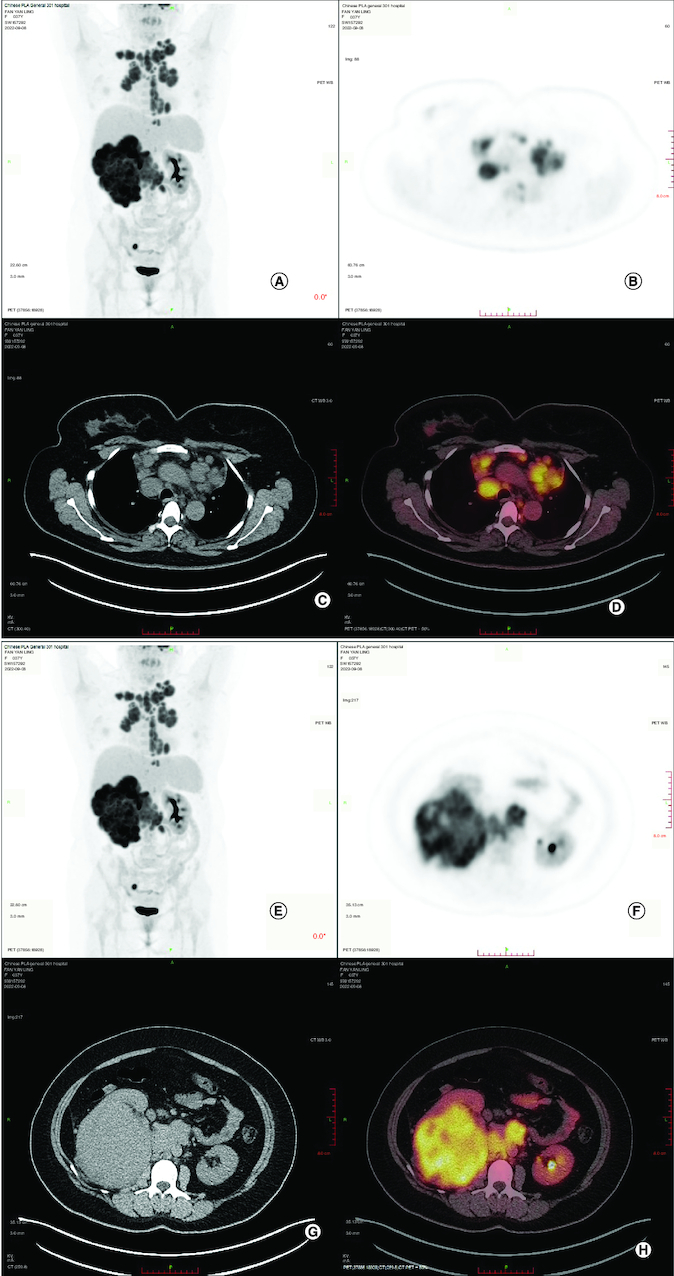
Whole body positron emission tomography-computed tomography before treatment. **(A–D)** Whole body PET-CT showed that enlarged hypermetabolic mediastinum lymph nodes(SUVmax:9.2). **(E–H)** Whole body PET-CT showed that a highly metabolic irregular mass in right kidney with heterogeneous density (120 mm*108 mm, SUVmax:15.2) and the border demarcation between the masses and the liver were indistinct. PET-CT: Positron emission tomography-computed tomography.

**Figure 2.**
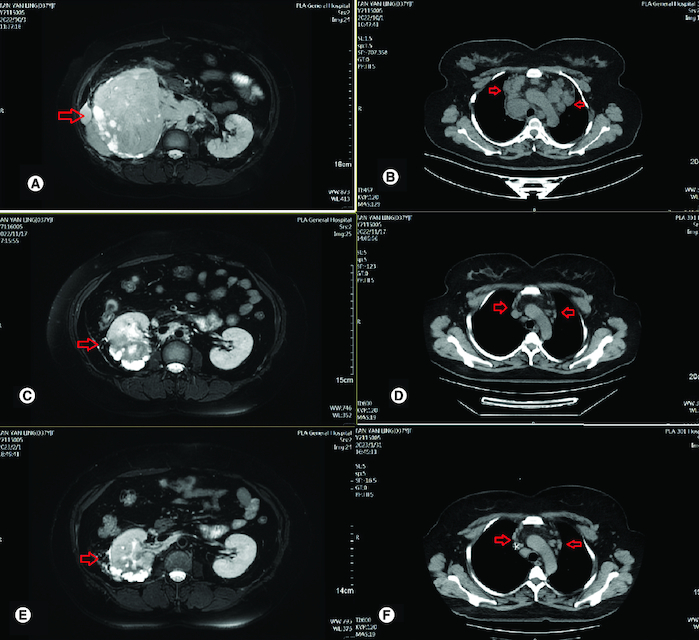
Radiological evaluation before and after treatment. **(A)** Abdominal MRI revealed irregular mass in right kidney with heterogeneous density and the soft tissue density shadows was seen in the inferior vena cava and right renal vein on 3 October 2022. **(B)** CT-SCAN revealed multiple enlarged mediastinum lymph node metastasis. **(C)** Abdominal MRI revealed irregular mass in right kidney with heterogeneous density significant reduction after two cycles of treatment. **(D)** CT-SCAN revealed significant mediastinum lymph node almost disappeared after two cycles treatment. **(E)** Abdominal MRI revealed irregular mass in right kidney with heterogeneous density continued reduction after four cycles of treatment. **(F)** CT-SCAN revealed significant mediastinum lymph node almost disappeared after four cycles of treatment.

**Figure 3.**
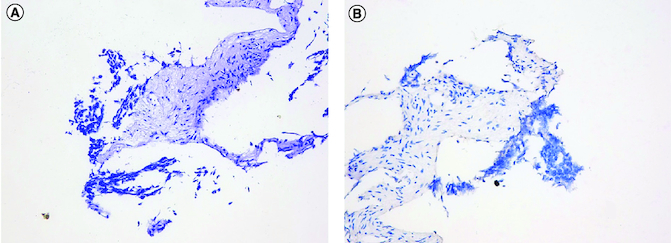
Staining images of PD-L1 expression (HE and IHC). **(A)** HE staining images of PD-L1 expression. **(B)** IHC staining images of PD-L1 expression. HE: Hematoxylin-eosin staining; IHC: Immunohistochemistry.

To confirm the diagnosis and identify metastatic sites in the whole body, whole body positron emission tomography-computed tomography (PET-CT) was performed, which showed a highly metabolic irregular mass in right kidney with heterogeneous density (120mm*108 mm, SUVmax:15.2) and the border demarcation between the masses and the liver were indistinct, PET-CT also indicated that several hypermetabolic lymph nodes, including left supraclavicular lymph nodes (SUVmax:10.2), bilateral subclavicular lymph node(SUVmax:10.2), mediastinum lymph nodes (SUVmax:9.2), hilar lymph nodes (SUVmax:9.2), retroperitoneal lymph nodes (SUVmax:10.3), pelvic lymph nodes (SUVmax:15.8), lymph nodes located around the lower esophagus and spine (SUVmax:9.2); the soft tissue density shadows was seen in the inferior vena cava and right renal vein, with increased metabolism (SUVmax:13.5) (Figure 1). Then, the patient underwent ultrasound-guided needle biopsy of tumor tissue in the right kidney, and the pathologic result showed small round blue cell tumors in the biopsy tissue, which was morphologically compatible with nephroblastoma, and the immunohistochemistry results showed positive expression of CK, WT1, BCL2, CD56, Vimentin, Syn, TLE-1 and SMA proteins, while Desmin, CD99, S100 and FLY-1 proteins were negative. The Ki-67 index was 70%, and PD-L1 (SP263) and Her-2 expressions were negative (Figure 3). Genetic test results showed *TP53* mutation (exon5 c.524G>A p.R175H). Based on COG staging system, the patient was diagnosed at stage IV. After discussion by a MDT with doctors of urology surgery, oncology, radiation oncology and interventional radiography, a consensus of systemic therapy was reached and radiation therapy can be given for well controlled conditions. Thus, a therapeutic strategy of chemotherapy (nab-paclitaxel plus carboplatin) combined with anti-PD-1 antibody (sintilimab) and VEGF-targeted therapy (bevacizumab) was developed for the patient, considering the synergistic effect of chemotherapy, anti-angiogenesis and immunotherapy. After two cycles of treatment, the patient obtained partial response with the mass in the right kidney significantly reduced and supraclavicular lymph nodes, bilateral subclavicular lymph nodes and mediastinum lymph nodes were almost invisible (Figure 2C & D). Only grade 2 myelosuppression occurred during the first two cycles of treatment. From October 2022 to January 2023, the patients received four cycles of treatment, and the assessment was still partial response (Figure 2E & E). Due to the invasion of the right renal vein and inferior vena cava after four cycles of treatment, the patient was still not suitable for surgery after discussion by a multidisciplinary team. Unfortunately, the patient was infected with COVID-19 and the immunotherapy was discontinued in case of pneumonia. Thereafter, the patient received two cycles of nab-paclitaxel plus bevacizumab as maintenance treatment. Up to the last follow-up time (April 2023), the patient achieved a PFS of 6 months without disease progression.

## Discussion

Wilms' tumor ranks as the most prevalent pediatric genitourinary malignancy, primarily afflicting children under the 5 years of age, with the highest incidence observed among those 3 to 4 years of age. Notably, there is no significant gender disparity in its incidence [7]. Conversely, in adults, the occurrence of Wilms' tumor is exceedingly rare, at just 0.2 cases per million per year. According to a study conducted by European Cancer Registries, the median age at which adults are diagnosed with Wilms' tumor is 34 years of age [6]. Approximately 1% of infants are born with nephrogenic rest cells within their kidneys, and these retained cells can potentially progress into Wilms' tumors [8]. Moreover, such progression is linked to the malfunction of the *WT-1* gene [9]. Studies have proved that the diffuse anaplasia in Wilms tumours with *TP53* mutations have poor outcome [10]. Our patient had a *TP53* mutation, however, unfortunately, due to insufficient tissue sampling, we were unable to further classify the pathology.

A retrospective analysis of the National Cancer Database suggested that the 5-year OS was significantly better in pediatric patients (<16 years) than young adult (16–35 years) and adult (>35 years) patients (93.1 vs 79.1 vs 78.8%) [11], as the majority of AWT was diagnosed with advanced stage.

Surgery is the most important treatment for Wilms' tumor. Over the last five decades, the management of Wilms' tumor in children has shown significant improvement. The 5-year survival rate for children treated has exceeded 85% [12]. Despite advances in surgical techniques and chemotherapy, the prognosis for AWT remains poor [3], and there is no standard treatment for AWT.

To share the experience of AWT treatment in our institution, we reported present case report. Most AWT patients received chemotherapy referring to treatment of pediatric Wilms' Tumor [1]. Given the rare occurrence of adult nephroblastoma, there is insufficient evidence to demonstrate that it can yield favorable outcomes when following pediatric protocols based on histological type and stage. Additionally, the toxicity associated with such treatments is higher in adults compared with children [13].

The most commonly used chemotherapy regimens, included doxorubicin, vincristine and actinomycin-D (VDA) [14.^15^]. The 4-year recurrence-free survival (RFS) and OS were 57.7 and 61.5%, respectively in pediatric patients receiving chemotherapy followed by radiation therapy, while there was limited clinical data for AWT [14]. Considering the significant toxicity, including neurotoxicity and hepatotoxicity [1,2], previous study reported that about 43% of AWT patients receiving vincristine and actinomycin-D suffered from severe neurotoxicity (grade 3–4) [4]. Therefore, novel treatment is needed to improve the outcomes of AWT. Currently, nab-paclitaxel plus carboplatin (Nab-PC) has been extensively studied in advanced non-small-cell lung cancer [16,17], breast cancer [18–20], esophageal cancer [21] and ovarian cancer [22]. A number of clinical studies showed that nab-paclitaxel and carboplatin have good effectiveness and safety. Nab-paclitaxel plus carboplatin revealed 33% overall response rate, and only 3% patients experienced peripheral neurotoxicity of grade 3/4 [23,24]. Therefore, nab-paclitaxel plus carboplatin might be effective in AWT with reduced toxicity, and was selected in our case.

Immunotherapy targeting PD-1/PD-L1 pathway has greatly changed the landscape of cancer treatment. The PD-L1 expression is frequently upregulated in Wilms' tumor. A pilot study suggested that an immune-engaged tumor microenvironment is present within Wilms' Tumor cells [25]. Another study showed that 65% of patients with Wilms' tumor demonstrated B7-H1 staining and 22% demonstrated staining diffusely [26]. Therefore, blocking the PD-1/PD-L1 pathway with immune checkpoint inhibitors may enhance the antitumor response in Wilms' tumor. A number of phase III clinical studies have demonstrated that PD-1 inhibitors combined with chemotherapy showed promoted anti-tumor efficacy than chemotherapy alone in NSCLC, ESCC, suggesting synergistic effect of chemotherapy combination with immunotherapy. Therefore, we choose chemotherapy combined with immunotherapy for the patient in present case.

VEGF is the key regulator of tumor angiogenesis by pro-angiogenic activities and inducing immunosuppression in the tumor microenvironment via accumulation of immature Treg, DC and MDSC [27,28]. Thus, anti-angiogenesis could augment response to immune checkpoint inhibitors, especially in immune-desert tumors. Anti-angiogenesis combined with PD-1/PD-L1 antibodies has showed promising anti-tumor efficacy in various cancers, such as liver cancer and renal cancer [10,28,29]. Therefore, immunotherapy combined with anti-angiogenesis might be effective in AWT.

Thus, the patient obtained nab-paclitaxel plus carboplatin combined with PD-1 antibody and anti-angiogenesis therapy in present case and achieved satisfactory effect.

## Conclusion

Our present case suggested that immunotherapy and anti-angiogenesis combined with chemotherapy is promising new approach for treating AWT. However, clinical trials and researches on molecular mechanisms are needed to confirm our findings and to determine the optimal combination of these therapies in the treatment of AWT.
